# A new asymmetry, an old toolkit: An emerging developmental enigma in appendage evolution

**DOI:** 10.1371/journal.pbio.3004078

**Published:** 2026-11-11

**Authors:** Caroline Schwaner, Tetsuya Nakamura

**Affiliations:** Department of Genetics, Rutgers, The State University of New Jersey, Piscataway, New Jersey, United States of America

## Abstract

Dorsoventral polarity is a key feature of paired appendages, yet its evolutionary origin is enigmatic as median fins, which predate paired appendages, are symmetric. A new study shows that lmx1b is dorsal in paired fin mesenchyme but posterior in median fins, implying genetic rewiring of an ancient toolkit.

## Teaser

Dorsoventral polarity is a key feature of paired appendages, yet its evolutionary origin is enigmatic as median fins, which predate paired appendages, are symmetric. A new PLOS Biology study shows *lmx1b* is dorsal in paired fin mesenchyme but posterior in median fins, implying genetic rewiring of an ancient toolkit.

Our hands provide a familiar example of dorsoventral (DV) asymmetry, with hair and nails on the dorsal surface and a hairless palm on the ventral side. Tetrapod (four-limbed vertebrates, including humans) limbs integrate dorsoventrally polarized components, including dorsal extensor and ventral flexor muscles, and dorsoventrally asymmetric bones and neurons, which support body weight and movement across diverse habitats. Despite the functional significance of DV asymmetry in limbs, the evolutionary origins of its underlying genetic program remain unclear. By contrast, decades of developmental studies have revealed that Engrailed-1 (En1) in the ventral ectoderm restricts Wnt7a to the dorsal ectoderm, where it induces Lmx1b in the dorsal mesenchyme to establish DV polarity [1–3]. Although less obvious, fish paired fins (the evolutionary antecedents of limbs) also exhibit DV asymmetry. The fin rays themselves can be dorsoventrally asymmetric, with dorsal and ventral hemitrichia, which each form one half of the fin ray, differing in morphology [4]. For example, the proximal ventral hemitrichia of pectoral fin rays possess flanges for fin muscle attachment. This skeletal asymmetry is accompanied by DV asymmetry of fin musculature, including morphologically distinct dorsal adductor and ventral abductor muscles that enable precise fin maneuverability. Mechanisms that establish DV asymmetry in paired fins, however, remain understudied.

Understanding the evolutionary origin of paired appendage DV regulation requires examining the evolutionary predecessors of paired fins. The lateral fin-fold theory, a long-standing hypothesis for the origin of paired fins, proposes that paired fins arose from elongated bilateral fin folds extending along the ventral body [5]. Because median fins (unpaired dorsal, anal, and caudal fins) predate paired fins, components of their ancestral developmental toolkit genes may have been co-opted for evolution of paired fins [6]. However, this scenario raises a fundamental problem. Unpaired fins are symmetric along the left-right (LR) axis, whereas paired fins protrude laterally from the body, involving a change from the original LR symmetry to DV polarity (Fig 1). In parallel, paired fins also acquired DV asymmetric morphology. A recent study compared limb patterning toolkit genes in ray-finned, cartilaginous, and jawless (which lack paired fins) fishes and suggested that *lmx1b* is expressed in the posterior mesenchyme of unpaired fins, but in the dorsal mesenchyme of paired fins [7]. Pharmacological experiments suggested that Hedgehog signaling in unpaired fins, but Wnt signaling in paired fins regulate *lmx1b* expression, implying that ancestral developmental components may have acquired new regulatory inputs for the evolution of DV asymmetry in paired fins.

**Fig 1:**
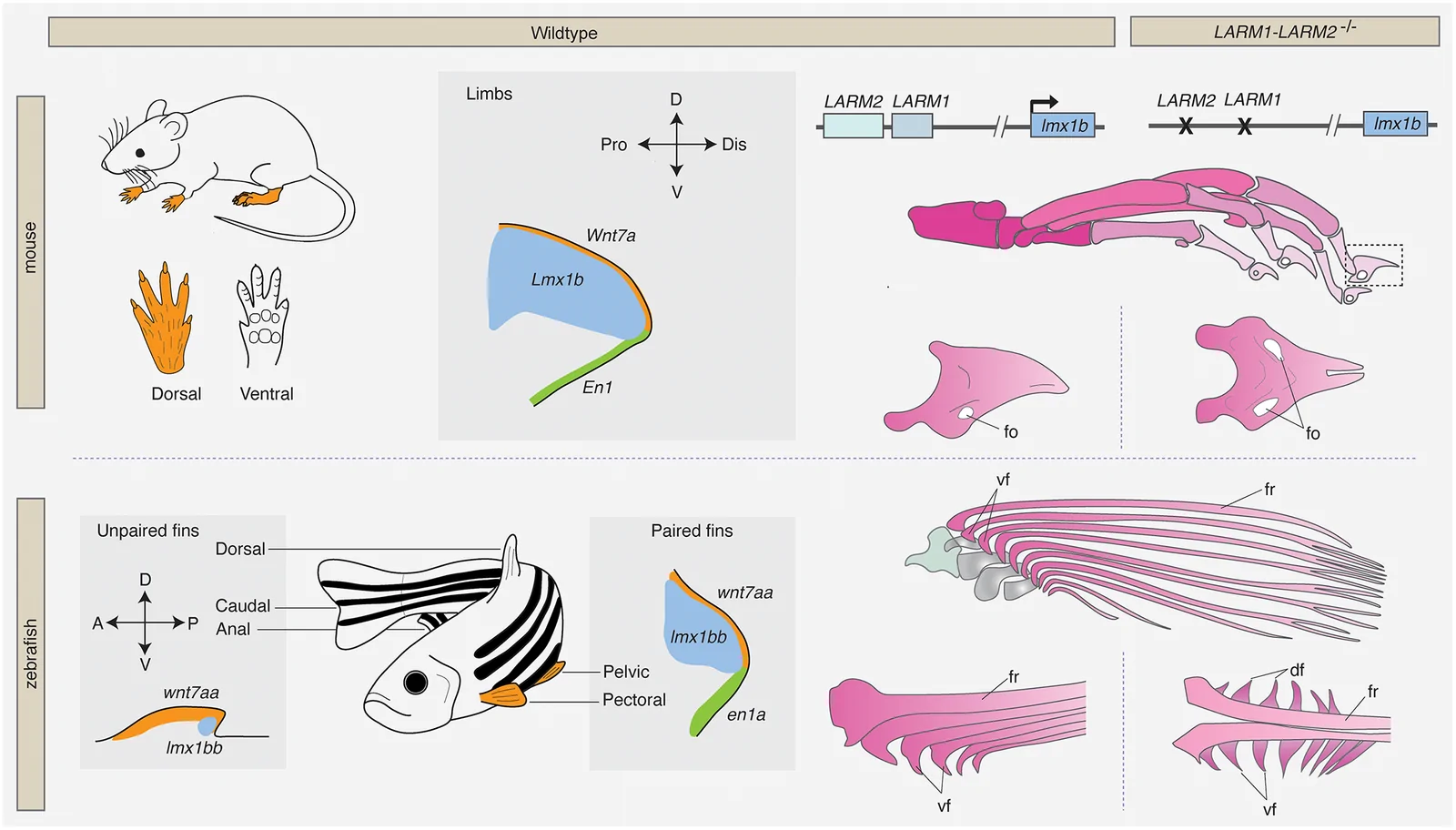
The genetic regulatory networks of the dorsoventral asymmetry in vertebrate appendages. The DV asymmetry of paired appendages (pectoral fins and limbs) is established by ectodermal *wnt7a* and *en1* and mesodermal *lmx1b*, while unpaired fins express *wnt7a* and *lmx1b* symmetrically across the midline. Deletions of both LARMs induce bi-ventralized appendages; digit tips of the hindlimb in mice and proximal fringes in zebrafish showed mirror-image phenotypes. For digit phenotype in mice, bony ventral foramen (fo) associated with the toe pad is magnified under the wildtype hindlimb skeleton. For pectoral fin phenotype, fringe phenotypes at the proximal fin rays are shown under the pectoral fin skeleton. Abbreviations: A, anterior; D, dorsal; df, dorsal fringe; Dis, distal; fr, fin ray; P, posterior; Pro, proximal; V, ventral; vf, ventral fringe; The skeletal phenotype of LARM knockout mice was drawn based on Haro and colleagues, 2021 [8].

In this issue, Dale and colleagues address this enigma by investigating the developmental regulation and function of canonical limb dorsal patterning genes, particularly *lmx1b,* across paired and unpaired fins [8]. They first showed that zebrafish paired fins have limb-comparable patterning, with *lmx1bb* (a duplicated *lmx1b* gene in teleosts) in dorsal mesenchyme, implying a conservation of spatial polarities of DV patterning genes between paired fins and limbs (Fig 1). Intriguingly, in the median fins of zebrafish, *lmx1bb* is also expressed but restricted to the posterior fin mesenchyme with a lateral symmetry. Comparative studies conducted by Dale and colleagues demonstrated that, in paddlefish, epaulet sharks, and elephant sharks, other canonical DV patterning genes, such as *wnt7a*, also possess dorsoventrally polarized expressions in paired fins but symmetric patterns in unpaired fins. Moreover, genetic cell lineage tracing in zebrafish demonstrated that *lmx1bb-*positive cells differentiate into osteoblasts of dorsal fin rays in paired fins but contribute symmetrically across the midline in median fins. These results suggest that components of the DV regulatory program in median fins may have been co-opted into paired fin developmental networks. However, the distinct expression domains of *lmx1b* between paired and unpaired fins suggest divergent regulatory logic.

Previous studies in mice identified two *cis-*regulatory modules, *LARM1* and *LARM2*, that drive *Lmx1b* expression in the dorsal mesenchyme of forelimbs [9]. Dale and colleagues found sequence conservation of *LARM1* among ray-finned and cartilaginous fishes, whereas *LARM2* conservation was restricted to the former, suggesting that at least one *LARM* was established in the last common ancestor of fishes with paired-fins and limbed-vertebrates. Deletions of both *LARM*s in zebrafish reduced *lmx1bb* enhancer activity in paired fins, but had no significant effect in median fins. Loss of both *LARM*s produced a bi-ventralized pectoral fin phenotype, in which the ventral fringes of fin rays were mirrored in dorsal fin rays (Fig 1), whereas median fins remained unaffected. Independent findings corroborate the functional significance of *Lmx1b* in paired fin development presented by Dale and colleagues [10]. Consistent with Dale and colleagues, disruption of *lmx1bb* and the *LARMs* led to a bi-ventralized pectoral fin phenotype, demonstrating that *lmx1bb* is the genetic determinant of dorsal identity in zebrafish paired fins. These studies suggest that evolution of *LARM*s co-opted *lmx1b* expression from a posterior domain in a LR symmetric appendage to a dorsal domain of paired appendages, establishing the novel DV polarity.

In mice, Lmx1b reinforces its own expression through a positive feedback loop with *LARM1* and *LARM2*, but the transcription factors that directly initiate *lmx1b* fin expression remain unknown. Further investigation of *lmx1b* regulation could reveal how Wnt and Hedgehog signaling induce *lmx1b* expression and were rewired to achieve DV polarity in paired fins. Furthermore, alongside the genetic rewiring of *lmx1b* regulation, the absence of *en1* in unpaired fins suggests that *en1* was recruited into a new ventral regulatory program during paired fin evolution. Systematic investigations of *en1 cis-*regulatory landscapes would also highlight rewiring of regulatory inputs and integration of *en1* into appendage developmental systems, which perhaps took place in parallel to the reprogramming of *lmx1b* controls. In limbs, En1 contributes to apical ectodermal ridge formation and restricts Wnt7a expression to the dorsal ectoderm through the regulation of tissue compaction [11]. A comparable function of *En1* in restricting *wnt7aa* to the dorsal pectoral fin was observed, yet the evolutionary origin of this function remains unresolved [10]. Overall, Dale and colleagues begin to reveal how paired fin DV asymmetry evolved through stepwise genetic rewiring of an ancestral developmental toolkit present in unpaired fins, illuminating how ancestral fins were transformed into the limbs we see today.
